# A Multicenter Retrospective Study Evaluating Distal Radial Access vs. Conventional Transradial or Transvenous Access for Endovascular Treatment of Malfunctioning Dialysis Fistulas

**DOI:** 10.3390/life14111382

**Published:** 2024-10-28

**Authors:** Roberto Minici, Massimo Venturini, Giuseppe Guzzardi, Federico Fontana, Andrea Coppola, Filippo Piacentino, Marco Spinetta, Davide Costa, Maria Chiara Brunese, Pasquale Guerriero, Biagio Apollonio, MGJR Research Team, Nicola De Rosi, Raffaele Serra, Domenico Laganà

**Affiliations:** 1 Radiology Unit, University Hospital Dulbecco, 88100 Catanzaro, Italy; derosinicola@gmail.com (N.D.R.); domenico.lagana@unicz.it (D.L.); 2 Diagnostic and Interventional Radiology Unit, ASST Settelaghi, Insubria University, 21100 Varese, Italy; massimo.venturini@uninsubria.it (M.V.); federico.fontana@uninsubria.it (F.F.); andrea.coppola@asst-settelaghi.it (A.C.); filippo.piacentino@asst-settelaghi.it (F.P.); 3 Imagerie Vasculaire et Interventionnelle, Centre Hospitalier Princesse Grace, 98000 Monaco City, Monaco; giuguzzardi@gmail.com; 4 Radiology Unit, Maggiore della Carità University Hospital, 28100 Novara, Italy; marcospinetta90@gmail.com; 5 Department of Law, Economics and Sociology, University “Magna Graecia” of Catanzaro, 88100 Catanzaro, Italy; davide.costa@unicz.it; 6 Department of Medicine and Health Sciences, University of Molise, 86100 Campobasso, Italy; mariachiarabrunese@gmail.com (M.C.B.); pasqualeguerriero@gmail.com (P.G.); 7 Department of Precision Medicine, University of Campania “L. Vanvitelli”, 81100 Naples, Italy; 8 Radiology Unit, San Timoteo Hospital, 86039 Termoli, Italy; bapollonio@sirm.org; 9 Vascular Surgery Unit, Department of Medical and Surgical Sciences, Magna Graecia University of Catanzaro, Dulbecco University Hospital, 88100 Catanzaro, Italy; rserra@unicz.it

**Keywords:** transradial access, transvenous access, endovascular management, malfunctioning dialysis fistulas, distal radial artery, hemodialysis fistula, radial artery, radial access, angioplasty

## Abstract

Background: This study aims to evaluate the feasibility, efficacy, and safety of distal transradial access (dRA) for the endovascular management of malfunctioning dialysis fistulas. This study also compares dRA with conventional access techniques, such as proximal radial and transvenous access, focusing on technical success, clinical outcomes, and vascular access site complications (VASCs). Methods: A retrospective multicenter study was conducted across four hospitals, including 292 patients treated between January 2019 and June 2024. Of these, 57 patients underwent dRA, and 235 received proximal radial or transvenous access. Key outcomes included technical success (successful completion of the procedure), clinical success (restoration of functional dialysis access), and complication rates. Data were collected on procedure times and complication profiles. Results: Technical success was achieved in 96.5% of patients undergoing dRA, compared to 98.3% in those receiving conventional access (*p* = 0.388). Clinical success was similar between groups (96.5% vs. 97%, *p* = 0.835). The overall complication rate was 10.5% for dRA and 8.5% for conventional access (*p* = 0.632). Cannulation time was longer for dRA (109.1 vs. 91.9 s, *p* < 0.001), but total procedure duration was comparable between the groups. No major complications were observed in either cohort, and improved post-procedure access flow rates were recorded in all patients. Conclusions: Distal transradial access is a feasible and effective approach for the endovascular management of malfunctioning dialysis fistulas, with outcomes comparable to conventional access techniques. It provides a safe alternative, particularly for patients with complex fistulas, while maintaining a low complication profile.

## 1. Introduction

Stenoses commonly threaten the patency of dialysis access, thus leading to dysfunction of mature arteriovenous fistulas (AVFs) [1]. Traditionally, percutaneous procedures typically involve making a venous puncture of the fistula for AVF access and subsequent treatment with balloon angioplasty to manage stenosis [2]. However, the effectiveness of this approach decreases when dealing with lesions distant from the puncture site, multiple concurrent lesions affecting the venous outflow, poor vein maturation in distal AVF, and numerous side branches between the anastomosis and venous access, with the arterial inflow poorly depicted despite tourniquet application [3,4,5].

While venous outflow is typically preferred, situations arise where arterial access becomes advantageous [4,5]. Transradial access (TRA) allows for the simultaneous treatment of multiple stenoses, some affecting venous outflow, and easily addresses juxta-anastomotic lesions, especially if a complex venous anatomy is noted. Furthermore, it facilitates comprehensive angiography, ensuring clear visualization of the entire AVF tract, while also avoiding brachial artery access and potential vascular access site complications (VASCs) to arterial inflow [3,6,7].

Recently, there has been a rise in the use of the distal radial artery (dRA) as an alternative vascular access point, offering a decreased risk in radial artery occlusion (RAO) compared to traditional proximal radial access (pRA) [8,9]. This advantage is particularly significant for patients requiring repeated endovascular procedures (e.g., percutaneous coronary intervention (PCI), transcatheter arterial chemoembolization (TACE), endovascular management of malfunctioning dialysis fistulas, etc.), due to which the cumulative risk of VASCs such as RAO becomes significant [9,10]. Furthermore, pRA may be impossible to perform when dealing with distal arteriovenous fistulas, and therefore may be very close to the conventional radial access site. In such cases, the dRA may have a pivotal role through the puncture of the radial artery at the anatomical snuffbox [11,12,13,14].

While the use of pRA for the endovascular management of malfunctioning dialysis fistulas is well consolidated in clinical practice thanks to numerous investigations that have demonstrated its efficacy and safety [4], the dRA has only been described in a few case reports or small case series [11,12,13,14]. The aim of our multicenter retrospective study is to assess the feasibility, efficacy, and safety of distal transradial access for the endovascular management of malfunctioning dialysis fistulas. Additionally, this study aims to compare distal transradial access with conventional vascular access sites (proximal radial and transvenous) to determine if the measures of feasibility, efficacy, and safety are comparable.

## 2. Materials and Methods

### 2.1. Study Design

This multi-center analysis includes data from the Mater-Domini Center at Dulbecco University Hospital (Catanzaro, Italy), Circolo Hospital (Varese, Italy), Maggiore della Carità University Hospital (Novara, Italy), and San Timoteo Hospital (Termoli, Italy). It examines prospectively collected data of consecutive patients who underwent endovascular management of dialysis fistulas between January 2019 and June 2024.

The inclusion criteria for the intervention group undergoing distal radial artery access site were as follows: (I) patients with anastomotic, juxta-anastomotic, venous, and/or arterial inflow strictures, requiring endovascular treatment for dialysis inefficacy; (II) patency of the distal radial artery; (III) patients age between 18 and 85 years; (IV) no prior endovascular procedures for malfunctioning AVF or vascular access performed in the same upper limb; (V) patency of the radiopalmar arch confirmed using the Barbeau test [15]; (VI) evaluation by a multidisciplinary team comprising nephrologists, vascular surgeons, and interventional radiologists. The exclusion criteria included the following: (I) non-palpable radial artery at the wrist or distal radial artery diameter less than 2 mm; (II) AVF thrombosis; (III) platelet count below 50,000/μL and/or international normalized ratio above 1.5; (IV) endovascular treatment for central venous stenosis or occlusion; (V) fistula that failed to mature; (VI) infected fistula; and (VII) impending rupture of a fistula-related aneurysm.

During the same study interval, data on patients undergoing endovascular management of AVFs with a venous or proximal radial artery access site were retrospectively evaluated to constitute a control group (i.e., also defined in the text as the “Conventional access Group”). The same indications and treatment techniques used in the distal radial access group were applied to the control group, except for access site hemostasis. The choice of vascular access depended on the preferences expressed by the interventional radiologist and on being subsequently endorsed during multidisciplinary discussions. In our institutions, transvenous access (TVA) is typically the primary choice, with TRA reserved for specific clinical scenarios. These scenarios include multiple stenoses, particularly those affecting venous outflow, complex venous anatomy, and juxta-anastomotic strictures that are difficult to manage with prior TVA. When radial artery access was chosen, the decision to use distal or proximal TRA was at the operator’s discretion, ensuring that the radial access site had a minimum diameter of 2 mm. Due to the retrospective nature of the study, ethical committee approval was not required. The research adhered to the ethical standards outlined in the Declaration of Helsinki. Written informed consent was obtained from all participants before beginning the endovascular procedure.

### 2.2. Treatment

**Figure 1:**
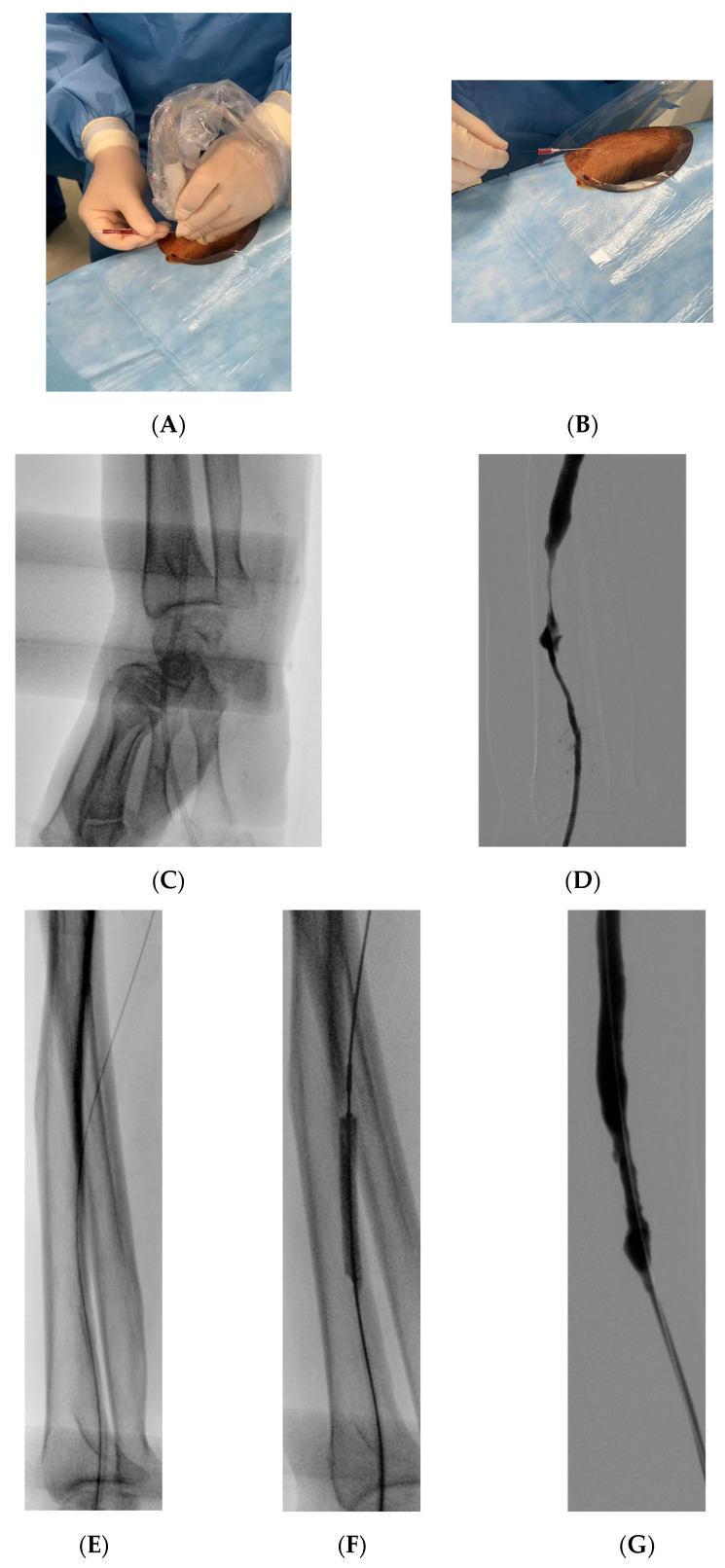
The patient’s forearm is positioned in a relaxed pronated position and a gauze roll is placed under the wrist to keep the hand flexed, thus exposing the anatomical snuff box. Arterial puncture of the distal radial artery is performed under ultrasound guidance with a 21-gauge micro-puncture needle, and a 0.014” guidewire is retrogradely advanced in the radial artery (**A**,**B**). Fluoroscopic check confirming the correct positioning of a 4 Fr introducer sheath through the distal radial artery (**C**). The angiogram revealed a significant stenosis in the mid-to-distal segment of the radial artery, just proximal to the radiocephalic dialysis fistula. Notably, the positioning of the introducer sheath in the distal radial artery provided sufficient room to fully advance the introducer into the vessel, ensuring its secure placement (**D**). The radial artery stenosis was successfully crossed with a 0.014” guidewire and treated with angioplasty using a 4 mm balloon, achieving an excellent final angiographic outcome (**E**–**G**).

A thorough arterial and venous color Doppler examination of the entire limb with the dialytic fistula was conducted within seven days before each intervention. The Barbeau test was utilized to evaluate the patency of the radiopalmar arch. The radial artery was punctured either at the conventional proximal site (a few centimeters above the styloid process) or at the distal site (at the anatomical snuffbox). After skin disinfection and local anesthesia, the radial artery was punctured under ultrasound guidance (Figure 1), and a 4 Fr or 5 Fr hydrophilic introducer sheath (Glidesheath Slender™; Terumo Corp, Tokyo, Japan) was inserted. A spasmolytic cocktail (200 mcg of Nitroglycerin, 2.5 mg of Verapamil, and 2500 IU of unfractionated heparin) was administered to prevent radial artery spasm and occlusion [16]. The procedure was performed by experienced consultant-grade interventional radiologists skilled in endovascular management of dysfunctional dialytic fistulas, using both TVA and TRA. Using a hydrophilic guide wire (Radifocus^TM^ Guide Wire M Standard Type 0.035” Angled; Terumo Corp, Tokyo, Japan) and a hydrophilic diagnostic catheter (Radifocus Glidecath; Terumo Corp, Tokyo, Japan), the arterial inflow was catheterized a few centimeters above the fistula site to perform a comprehensive diagnostic angiography, covering both the inflow and outflow segments. TVA was obtained through an ultrasound-guided puncture of the venous outflow, followed by angiographies before and after applying a tourniquet to occlude the venous outflow proximal to the introducer site. Target stenoses were then treated with fistuloplasty as indicated, using standard high-pressure balloons in accordance with KDOQI Clinical Practice Guidelines [17]. Finally, a completion angiography was performed from the arterial inflow. Patent hemostasis at the access site was achieved using a TR Band (TR Band^®^; Terumo Corp, Tokyo, Japan) for proximal TRA or compressive bandaging for distal transradial or TVA [18]. The TR Band was removed approximately four hours later, after confirming hemostasis at the radial access site. VASCs, including radial artery patency, were evaluated at the patient’s discharge and four weeks post-treatment, using both clinical examination and Doppler ultrasound.

### 2.3. Outcomes and Definitions

The primary efficacy endpoint is the rate of technical success. The secondary efficacy endpoint includes the clinical success rate. The primary safety endpoint is defined by the VASC rate. Procedure time was set as the primary feasibility endpoint.

Patients who underwent distal TRA are included in the “Distal radial group”, whereas those whose vascular access was through the venous outflow or the proximal radial artery are categorized in the “Conventional access group”. Dialytic fistulas were considered to be malfunctioning based on clinical indicators such as repeated needle clotting, difficulties with needle insertion, extended bleeding times post-needle removal, limb swelling, reduced access flow (below 500 mL/min), high recirculation rates (over 15%), lowered blood flow rates, increased venous pressure, and other signs of reduced dialysis efficiency as determined by a nephrologist, along with stenoses of 50% or more as evaluated by a sonographer. AVF failure was defined as persistent fistula dysfunction necessitating surgical revision, creation of a new fistula, or central venous catheter placement. Fistulas were classified by their anatomical site into the radiocephalic, brachiocephalic, or brachial artery to transposed basilic vein (brachiobasilic), and other less common types [19]. Lesion locations were described similarly to Clark et al. and Shamimi-Noori et al. [12,20]. Lesions were categorized as affecting the arterial inflow segment, the arteriovenous anastomosis, within 2 cm of the anastomosis (juxta-anastomotic), or the venous outflow segment (more than 2 cm from the anastomosis). For multiple stenoses, the most severe one was used for classification. Patency (primary, assisted primary, and secondary) was evaluated according to the criteria by Huijbregts et al. [21]. Distal radial access refers to accessing the distal part of the radial artery at the anatomical snuffbox, as outlined by Kiemeneij [8]. Proximal radial access was performed a few centimeters above the styloid process. Conversion to another vascular access site, termed “conversion rate”, was documented when needed to complete the endovascular treatment [9]. The number of punctures was calculated considering each time the needle was pulled out of the skin after its insertion. Sheath upgrading, necessary for using a larger-diameter catheter for angioplasty, was noted when upgrading from a 4–5 Fr to a 6 Fr sheath was required. Radial artery spasm (RAS) and radial artery occlusion (RAO) were identified via angiography and Doppler ultrasound, respectively. Major bleeding was defined by a hemoglobin decrease of more than 3 g/dL [22,23]. Technical success was defined as achieving less than 30% residual stenosis. Clinical success was indicated by the patient’s ability to resume effective dialysis using the double-needle technique. Procedure-related complications were classified following the CIRSE Classification System for Complications [24], with significant complications being those of Grade 4 or higher. Definitions followed the standards of the Society for Vascular Surgery [23], the KDOQI Clinical Practice Guidelines for Vascular Access [14], and other previous studies [9,25,26], unless otherwise specified.

### 2.4. Statistical Analysis

Data management was conducted using an Excel spreadsheet (version 16.67 for Mac; Microsoft Inc., Redmond, WA, USA), and statistical analyses were performed with SPSS software (version 22 for Windows; SPSS Inc., Chicago, IL, USA). Normality of data was assessed using the Kolmogorov–Smirnov and Shapiro–Wilk tests [27,28]. Categorical variables are reported as frequencies (percentage) [29], while continuous data are presented as mean ± standard deviation if normally distributed, or as median (interquartile range: 25th and 75th percentiles—IQR) if not [30,31]. Statistical differences were examined using an unpaired Student’s t-test for continuous normally distributed data, the Chi-squared/Fisher’s exact test for categorical data, and the Mann–Whitney test for continuous data that were not normally distributed, as appropriate [32,33]. Patients’ data were censored at the end of the follow-up period, which extended to 31 March 2024, a period of 12 months post-intervention, at study discontinuation, upon definitive abandonment of a malfunctioning fistula, or in the event of patient death. Time-dependent outcomes were evaluated using a Kaplan–Meier survival analysis, with comparisons made via a log-rank test [34]. To ensure that the assumption of independent censoring was upheld, clinical evaluations and telephone follow-ups were conducted for patients who withdrew from the study. This approach helped to mitigate bias associated with time-dependent data. For all statistical tests, a *p*-value of <0.05 was deemed to be significant.

## 3. Results

### 3.1. Study Population

This study included 292 patients, with 235 undergoing conventional access (i.e., proximal radial or transvenous access) (Group 1) and 57 using distal radial access (Group 2). Age was similar between groups (66.7 years, *p* = 0.916), as was sex distribution (conventional: 37.9% female, distal: 36.8% female, *p* = 0.885). Hypertension rates were comparable (56.6% in the conventional group vs. 52.6% in the distal group, *p* = 0.589). Prevalence of cerebrovascular disease (28.9% vs. 21.1%, *p* = 0.231) and coronary artery disease (40.9% vs. 36.8%, *p* = 0.580) were similar between groups. Smoking history and current smoking rates were also comparable. Diabetes prevalence was 46% in the conventional group and 42.1% in the distal group (*p* = 0.600). Platelet counts, INR and aPTT values, as well as hyperlipidemia, coagulopathy, antiplatelet, and anticoagulant therapy rates, were similar across groups. Baseline demographic data are summarized in Table 1, indicating a well-matched distribution between the two groups.

### 3.2. Procedure Data

Table 2 compares procedural data between conventional access (Group 1) and distal access (Group 2) groups. There were no significant differences between the groups regarding fistula types or side distribution. However, significant differences were observed in several areas. Conventional access had a higher percentage of shorter stenosis lengths (less than 3 cm) compared to distal access (40.4% vs. 15.8%, *p* < 0.001) and fewer cases with longer stenosis (i.e., greater than 5 cm). Regarding stenosis location, conventional access had more venous stenoses compared to distal access (36.3% vs. 42.1%, *p* < 0.001). The number of punctures required was lower in the conventional access group compared to the distal group (1.15 vs. 1.45, *p* < 0.001). Cannulation time was also shorter for conventional access compared to distal access (91.9 vs. 109.1 s, *p* < 0.001), despite the overall procedure duration being consistent between groups. In terms of introducer sheath size, distal access utilized more 5 Fr sheaths compared to conventional access (84.2% vs. 71.9%, *p* = 0.014). Finally, conventional access used a higher contrast volume than distal access (50.3 mL vs. 40.8 mL, *p* < 0.001). Other factors such as fluoroscopy time, cumulative air kerma, and dose area product were similar between the two groups. Rates for introducer sheath upgrades and vascular access site conversions were also comparable. Interestingly, in two patients suffering from multiple venous stenoses, conversion to a venous access was needed to address a stenosis of the upper tract of the cephalic vein that was not crossable with a dRA due to the tightness of the stenosis and its long distance from the access site. Moreover, two cases of transvenous access required an additional arterial access to cross a tight anastomotic lesion, thus using a through-and-through technique.

### 3.3. Efficacy and Safety Outcomes

Technical and clinical success rates were high and comparable between the two access groups. Technical success was achieved in 97.9% of patients overall, with 98.3% for conventional access and 96.5% for distal radial access (*p* = 0.388). Clinical success rates were 96.9% in total, with 97% for conventional access and 96.5% for distal radial access (*p* = 0.835). Post-procedure access flow rates and average increases in flow rates were similar between groups, with an average post-procedure flow rate of 1050.7 mL/min for conventional access and 984.5 mL/min for distal radial access (*p* = 0.458). The overall procedure-related complication rate was 8.9%, with 8.5% for conventional access and 10.5% for distal radial access (*p* = 0.632). Vascular access site complications occurred in 4.7% of patients for conventional access and 8.8% for distal radial access (*p* = 0.223). The types of vascular access site complications were similar between groups. In terms of CIRSE classification, 91.1% of complications were classified as none, 8.9% as minor, and none as major. These proportions were comparable between the two access groups (*p* = 0.632). No surgical interventions were required for complications in any patients, and the need for medical or percutaneous treatments was also similar across both groups. Details are given in Table 3.

## 4. Discussion

The primary findings of our study are as follows:

- Distal radial access has recently emerged as an alternative vascular access site to proximal radial access [8]. While its use is well-established in interventional cardiology [35], its application in interventional radiology remains limited [9], with only a few case reports and series documenting its role in the endovascular management of AVFs [11,12,13,14]. Although the reduced risk of RAO is widely recognized as its main advantage [8], our experience has confirmed an additional benefit in its application for AVFs. Specifically, it allows for the treatment of very distal radiocephalic fistulas via arterial access without utilizing the arterial inflow as the vascular access site. This approach helps prevent vascular access site complications that could potentially damage the inflow or the anastomotic chamber.
- Distal radial access is effective for the endovascular management of malfunctioning fistulas, demonstrating a technical and clinical success rate comparable to conventional vascular access sites (proximal radial or transvenous). Although the allocation to the two groups was not randomized, arterial access is often chosen in more challenging scenarios (e.g., difficult-to-cross juxta-anastomotic stenoses, multiple venous outflow stenoses, complex venous anatomy, etc.) compared to simpler focal stenoses usually addressed via TVA. Therefore, we believe this selection bias did not significantly affect the observed efficacy outcomes.
- Distal radial access is safe, with a low VASC rate comparable to other vascular access sites. This finding may be influenced by the operators’ experience and strict adherence to stringent inclusion criteria (e.g., ultrasound-guided puncture, vessel diameter of at least 2 mm, etc.). The feasibility of distal radial access is good in a real-world scenario. Despite a longer initial cannulation time, the overall procedural time is similar due to easier and immediate angiography of the entire AVF compared to TVA.

To appreciate the benefits of using the distal radial artery (dRA) for endovascular management of dialysis fistulas, it is essential to understand the anatomical and pathophysiological rationale behind it. The use of the dRA as an access site for endovascular procedures was first described by Kiemeneij et al. in 2017 for coronary angiography [8]. Unlike the traditional puncture site located proximal to the radial styloid process, the dRA is typically accessed at the anatomical snuffbox, a triangular depression on the back of the hand bordered laterally by the abductor pollicis longus and extensor pollicis brevis tendons, and medially by the extensor pollicis longus tendon, with the floor formed by the scaphoid and trapezium bones [36]. The point of vascular access-site puncture, sheath introduction, and subsequent compression for hemostasis is the area at the highest risk of thrombosis and occlusion [37]. If thrombosis occurs at the conventional proximal radial cannulation site, it can extend retrograde since there are no collateral circles to maintain adequate antegrade flow in the radial artery. Conversely, if thrombosis occurs at the distal radial artery, hand circulation is maintained because the obstruction to flow is beyond the origin of the superficial palmar branch, thus preventing blood stasis during hemostasis and proximal thrombus growth [38,39]. Hence, dRA is associated with a reduced incidence of RAO compared to pRA [40,41]. Preventing radial artery occlusion is particularly important to ensure the artery’s reuse in clinical scenarios involving repeated endovascular procedures (TACEs, PCIs, fistuloplasties, etc.) or to preserve the radial artery for potential future coronary artery bypass grafting. In the context of AVFs, preventing RAO not only ensures the reuse of vascular access, but also avoids potential issues within the arterial inflow caused by proximal thrombus extension. Additional advantages include shorter time to hemostasis, improved operator and patient comfort, and applicability to patients with orthopedic conditions (e.g., frozen shoulder) that limit wrist supination [8]. Interestingly, our experience highlighted an additional advantage of dRA, beyond those reported in the literature for general endovascular purposes. This advantage pertains specifically to dRA application in AVFs, allowing for the treatment of very distal radiocephalic fistulas via arterial access without utilizing the arterial inflow as the vascular access site. Certain clinical scenarios favor arterial access over TVA (e.g., difficult-to-cross juxta-anastomotic stenoses, multiple venous outflow stenoses, poor vein maturation in distal AVF, complex venous anatomy, poor depiction of arterial inflow, etc.) [7]. Shamini-Noori et al. compared the transbrachial access and the TRA, highlighting fewer access site punctures, higher rates of clinical and technical success, superior primary patency, and improved assisted primary patency at 12 months for TRA [12]. Furthermore, transbrachial arterial access is associated with a significantly higher VASC rate compared to transradial access [12,42]. This is crucial not only due to the complications themselves, but also because the vascular access site coincides with the arterial inflow, which can potentially affect the fistula. For instance, major VASCs related to transbrachial arterial access often involve hemorrhage, requiring prolonged compression to achieve hemostasis [43], thereby increasing the risk of thrombosis at the anastomotic chamber. While the proximal radial artery is another possible arterial access site, it is not feasible for very distal radiocephalic fistulas [11,12]. Watanabe et al. measured the average distance between the anastomotic chamber and the conventional proximal radial access site in 12 distal AVFs treated with dRA, reporting an average of 2.2 cm [13]. This highlights the difficulty of positioning sheaths and maneuvering catheters and guidewires when using proximal radial access in distal forearm AVFs. Therefore, the distal radial artery offers a unique advantage in the realm of AVFs; it allows for the treatment of distal radiocephalic fistulas via arterial access [11], fostering the manipulation of catheters and guidewires and without using the arterial inflow as the access site, thereby preventing possible VASCs affecting the arterial inflow and the fistula itself.

Distal radial access is effective for the endovascular management of malfunctioning fistulas, demonstrating a technical and clinical success rate comparable to conventional vascular access sites (proximal radial or transvenous). Hull et al. reviewed 68 cases of endovascular management of radiocephalic fistulas, comparing outcomes between dRA access and direct fistula puncture. They reported a 100% technical success rate for both dRA and direct fistula access [14]. Similarly, Watanabe et al. successfully treated all 12 cases of AVFs using dRA in their 2020 case series [13]. Recently, Prismadani et al. presented a case report of a distal radiocephalic fistula thrombosis with proximal radial artery stenosis, effectively treated via dRA [11]. In our investigation, all patients exhibited a significant improvement in access flow rate, ensuring effective dialysis (namely, 100% clinical success rate). Interestingly, our retrospective analysis is the first multicenter study with a large sample size to compare dRA with conventional vascular access methods (i.e., TVA or pRA). Moreover, our results align with other published studies, demonstrating the effectiveness of dRA for managing malfunctioning dialysis fistulas. The dRA offers an alternative vascular access to pRA, which can be successfully used in specific clinical scenarios needing arterial access.

Distal radial access is safe, with a low VASC rate comparable to other vascular access sites. Hull et al. observed no major complications in 17 radiocephalic fistulas treated with dRA or in 51 treated with direct fistula puncture [14]. Watanabe et al. also reported no cases of RAO in their series of 12 AVFs treated using dRA [13]. Previous meta-analyses by Izumida and Liang on dRA for cardiac catheterization reported an incidence of RAO of approximately 1.4% and 1.7%, respectively [40,44]. In our experience, we did not encounter any major complications requiring subsequent surgical or endovascular treatment or which resulted in a deterioration of the patient’s general clinical condition.

Few instances of RAO have been recorded, which remained clinically inconsequential. Additionally, the safety outcomes we observed are consistent with the literature data on the use of dRA for endovascular procedures in other clinical settings. The incidence of neuropathy, pain, and access site infections is very low. Rarely, pseudoaneurysms, dissections, and arteriovenous fistulas have been reported [45,46]. The most common complication is access site hematoma, typically small and clinically insignificant [9,40,44]. Major bleeding is unusual thanks to the trapezium and scaphoid bones forming a bone base fostering hemostasis [47]. Therefore, our results, combined with existing safety data, support the safety of dRA for the endovascular management of AVFs.

The feasibility of dRA is excellent, given the lack of significant differences in procedural time compared to the conventional vascular access group. However, the cannulation time was significantly longer for dRA; therefore, it can be speculated that the overall procedural time was similar, likely due to the easier execution of arterial angiography, which provided better visualization and faster crossing of the culprit lesion. Similarly, in a recent study of 68 radiocephalic fistulas, the mean procedure times for snuffbox radial artery access and direct fistula access were not significantly different (29.1 min vs. 26.8 min, *p* < 0.57) [14]. Watanabe et al. also reported an average fluoroscopy time of 11 min in 12 AVFs treated with dRA [13]. Interestingly, Hull et al. recorded an average dRA diameter of 2.79 mm in 17 radiocephalic fistulas, similar to the mean proximal radial artery diameter of 2.78 mm [14]. This suggests that the average dRA diameter generally allows for the smooth insertion of 5 Fr sheaths and can accommodate upgrades to 6 Fr sheaths for larger balloon PTA or 7 Fr sheaths for thrombectomy devices (e.g., the 7 Fr Terumo Glidesheath Slender Sheath has an outer diameter of 2.45 mm). Our experience, marked by a very low conversion rate, indicates that switching to another vascular access site to use larger devices is quite rare. This finding aligns with previous anatomical studies of the radial artery, which have shown minimal diameter differences between the distal and proximal radial artery, not affecting the choice of sheath [48]. Previously, Rahmatzadeh et al. reported a 46.7% upgrade to a 6 Fr introducer in their cases treated via radial access [6], whereas our experience showed a significantly lower upgrade rate. This difference could be attributed to the exclusion of isolated central venous stenoses requiring large-diameter PTA balloons and technological advancements that have enabled the use of low-profile larger caliber PTA balloons compatible with 5 French introducers (e.g., Sterling balloon catheter, Boston Scientific Corporation, Natick, MA, USA). Finally, the feasibility of dRA is influenced by preoperative patient selection, confirming the patency of the radial–ulnar pathway with the Barbeau test [15] and ensuring that a sufficient dRA diameter to maintain an artery/sheath ratio >1, thus decreasing endothelial damage [49], is mandatory to minimize complications.

Several limitations associated with dRA should be acknowledged: (1) anatomical constraints (e.g., radial artery caliber measuring less than 2 mm) that restrict its application [50], as well as the use of large bore sheaths needed to address central venous stenoses with large-diameter PTA balloons or vascular ruptures with covered grafts. In some cases, the radial artery’s diameter allows for the placement of 8 French introducers. However, this comes with an increased risk of vascular injury and RAO [51,52]. (2) A longer cannulation time, which may incur biological costs in urgent scenarios until proficiency is gained (rare scenario for AVFs). (3) dRA diameter assessments and ultrasound-guided punctures of the access site are advisable. Therefore, the operator must possess at least basic ultrasound skills, as a one-size-fits-all approach is not advisable. (4) The potential impact on the scaphoid bone’s blood supply remains uncertain, given that the scaphoid bone is primarily vascularized by lateral and distal branches of the radial artery. (5) Anatomical factors, such as a radial loop, acute angulation at the anastomosis, a high origin of the radial artery, or stenosis, hamper the manipulation of catheters and guidewires [9,12,35].

Our study’s main limitations are its retrospective design, lack of randomization, and the extensive experience of our operators with TRA procedures. TRA requires a learning curve, and complications are less frequent in experienced centers [53], which could limit the generalizability of our findings. The careful attention to procedural details may have also contributed to the reduced VASC rate, potentially limiting applicability to less-rigorous clinical settings. Additionally, different criteria for selecting radial access versus TVA might introduce selection bias, although we believe this did not impact outcomes, as radial access was used in more challenging scenarios. Lastly, long-term outcomes were not assessed.

## 5. Conclusions

In conclusion, our report highlighted the efficacy, safety, and feasibility of distal radial access as a vascular access site for the endovascular management of malfunctioning dialysis fistulas through a multicenter retrospective analysis with a large sample size. Although transvenous access remains the first choice, there are specific clinical scenarios where transarterial access becomes preferable, and the distal radial artery is a viable alternative to the proximal radial artery and brachial artery, avoiding the use of the arterial inflow as a vascular access site. Adhering to certain technical precautions minimizes the occurrence of vascular access site complications.

## Figures and Tables

**Table 1:** Baseline demographic data.

Variables	All Patients (n = 292)			
		Group 1 Proximal Radial or Transvenous Access (n = 235)	Group 2 Distal Radial Access (n = 57)	*p*-Value
Age (years)	66.7 (±11.5)	66.7 (±11.5)	66.7 (±11.6)	0.916
Sex (F/M)	110 (37.7%)/182 (62.3%)	89 (37.9%)/146 (62.1%)	21 (36.8%)/36 (63.2%)	0.885
Hypertension	163 (55.8%)	133 (56.6%)	30 (52.6%)	0.589
Cerebrovascular disease	80 (27.4%)	68 (28.9%)	12 (21.1%)	0.231
Coronary artery disease	117 (40.1%)	96 (40.9%)	21 (36.8%)	0.580
Smoking history	195 (66.8%)	160 (68.1%)	35 (61.4%)	0.337
Current smoker	121 (41.4%)	97 (41.3%)	24 (42.1%)	0.909
Diabetes	132 (45.2%)	108 (46%)	24 (42.1%)	0.600
Hyperlipidemia	183 (62.7%)	144 (61.3%)	39 (68.4%)	0.317
INR	1.34 (±0.3)	1.35 (±0.3)	1.32 (±0.3)	0.446
aPTT (s)	39 (±5.7)	39.1 (±5.7)	38.7 (±5.9)	0.762
Platelet count (No. ×10^3^/μL)	333.6 (±127.5)	328.8 (±128.3)	353.3 (±123.2)	0.087
Coagulopathy	119 (40.8%)	92 (39.1%)	27 (47.4%)	0.257
Antiplatelet therapy	154 (52.7%)	124 (52.8%)	30 (52.6%)	0.985
Anticoagulant therapy	134 (45.9%)	110 (46.8%)	24 (42.1%)	0.523

Abbreviations: μL: microliter; aPTT: activated partial thromboplastin time; F: female; INR: international normalized ratio; M: male; s: seconds.

**Table 2:** Procedure data.

Variables	All Patients (n = 292)			
		Group 1 Proximal Radial or Transvenous Access (n = 235)	Group 2 Distal Radial Access (n = 57)	*p*-Value
Fistula: - Radiocephalic - Brachiocephalic - Brachiobasilic - Others (Gracz, Prosthetic, etc.)	75 (25.7%) 101 (34.6%) 70 (24%) 46 (15.8%)	60 (25.5%) 82 (34.9%) 56 (23.8%) 37 (15.7%)	15 (26.3%) 19 (33.3%) 14 (24.6%) 9 (15.8%)	0.997
Side (Right/Left)	102 (34.9%)/190 (65.1%)	82 (34.9%)/153 (65.1%)	20 (35.1%)/37 (64.9%)	0.978
Pre-procedure access flow rate (mL/min)	559.6 (±269.2)	571.6 (±298.6)	509.9 (±311.8)	0.781
Stenosis length: - <3 cm - 3–5 cm - >5 cm	104 (35.6%) 118 (40.4%) 70 (24%)	95 (40.4%) 82 (34.9%) 58 (24.7%)	9 (15.8%) 36 (63.2%) 12 (21.1%)	<0.001
Stenosis location: - Artery - Anastomotic - Juxta-anastomotic - Venous	19 (6.5%) 75 (25.7%) 92 (31.5%) 106 (36.3%)	4 (1.7%) 66 (28.1%) 83 (35.3%) 82 (34.9%)	15 (26.3%) 9 (15.8%) 9 (15.8%) 24 (42.1%)	<0.001
Number of access site punctures	1.22 (±0.55)	1.15 (±0.45)	1.45 (±0.83)	<0.001
Successful cannulation and sheath introduction	292 (100%)	235 (100%)	57 (100%)	NA
Cannulation time (s)	95.3 (±32.4)	91.9 (±32.5)	109.1 (±28)	<0.001
Introducer sheath size: - 4 Fr - 5 Fr - 6 Fr - ≥7 Fr	37 (12.7%) 217 (74.3%) 30 (10.3%) 8 (2.7%)	28 (11.9%) 169 (71.9%) 30 (12.8%) 8 (3.4%)	9 (15.8%) 48 (84.2%) 0 (0%) 0 (0%)	0.014
Introducer sheath upgrade	11 (3.8%)	8 (3.4%)	3 (5.3%)	0.508
Vascular access site conversion	5 (1.7%)	2 (0.9%)	2 (3.5%)	0.362
Contrast volume (mL)	48.4 (±16)	50.3 (±16.2)	40.8 (±12.5)	<0.001
Procedure duration (min)	46.5 (±14.4)	45.9 (±14)	48.7 (±15.8)	0.485
Fluoroscopy time (min)	9.7 (±3.6)	9.7 (±3.5)	9.8 (±4)	0.557
Cumulative air kerma (mGy)	189.3 (±65.6)	191 (±66.6)	182.2 (±61.6)	0.089
Dose area product (DAP) (Gy/cm^2^)	22.2 (±8.6)	22.4 (±8.6)	21.6 (±8.8)	0.132

Abbreviations: cm: centimeters; Gy: gray; min: minutes; mL: milliliter; s: seconds.

**Table 3:** Outcome data.

Variables	All Patients (n = 292)			
		Group 1 Proximal Radial or Transvenous Access (n = 235)	Group 2 Distal Radial Access (n = 57)	*p*-Value
Technical success	286 (97.9%)	231 (98.3%)	55 (96.5%)	0.388
Clinical success	283 (96.9%)	228 (97%)	55 (96.5%)	0.835
Post-procedure access flow rate (mL/min)	1037.8 (±254.2)	1050.7 (±278.7)	984.5 (±86.3)	0.458
Average increase in access flow rate (mL/min)	478.2 (±124.2)	479.1 (±133.6)	474.6 (±74.7)	0.891
Procedure-related complication rate	26 (8.9%)	20 (8.5%)	6 (10.5%)	0.632
Vascular access site complication rate	16 (5.5%)	11 (4.7%)	5 (8.8%)	0.223
Vascular access site complication: - None - Hematoma - Access site spasm - Access site occlusion	276 (94.5%) 12 (4.1%) 1 (0.3%) 3 (1.1%)	224 (95.3%) 9 (3.8%) 0 (0%) 2 (0.9%)	52 (91.2%) 3 (5.3%) 1 (1.8%) 1 (1.8%)	0.187
Procedure-related complications (CIRSE classification): - None - Minor (grade 1–2–3) - Major (grade 4–5–6)	266 (91.1%) 26 (8.9%) 0 (0%)	215 (91.5%) 20 (8.5%) 0 (0%)	51 (89.5%) 6 (10.5%) 0 (0%)	0.632
Required treatment for complications: - None - Medical - Percutaneous - Surgical	266 (91.1%) 26 (8.9%) 0 (0%) 0 (0%)	215 (91.5%) 20 (8.5%) 0 (0%) 0 (0%)	51 (89.5%) 6 (10.5%) 0 (0%) 0 (0%)	0.632

Abbreviations: min: minutes; mL: milliliter.

## Data Availability

The data presented in this study are available on request from the corresponding author. The data are not publicly available due to privacy issues.

## Abbreviations

AVF: arteriovenous fistula; BB: brachiobasilic; BC: brachiocephalic; CI: confidence interval; cTRA: conventional transradial access; DAP: dose-area product; dRA: distal radial artery; DSA: digital subtraction angiography; dTRA: distal transradial access; KDOQI: Kidney Dialysis Outcomes Quality Initiative; NKF: National Kidney Foundation; PCI: percutaneous coronary intervention; pRA: proximal radial access; RAO: radial artery occlusion; RAS: radial artery spasm; RC: radiocephalic; TACE: transcatheter arterial chemoembolization; TRA: transradial access; TVA: transvenous access; US: ultrasound; VASCs: vascular access site complications.
